# A Lab Assembled Microcontroller-Based Sensor Module for Continuous Oxygen Measurement in Portable Hypoxia Chambers

**DOI:** 10.1371/journal.pone.0148923

**Published:** 2016-02-10

**Authors:** Saroj P. Mathupala, Sam Kiousis, Nicholas J. Szerlip

**Affiliations:** 1 Department of Neurosurgery and Karmanos Cancer Institute, Wayne State University School of Medicine, Detroit, Michigan, United States of America; 2 Department of Neurosurgery, University of Michigan Medical School, Ann Arbor, Michigan, United States of America; University of Nebraska Medical Center, UNITED STATES

## Abstract

**Background:**

Hypoxia-based cell culture experiments are routine and essential components of *in vitro* cancer research. Most laboratories use low-cost portable modular chambers to achieve hypoxic conditions for cell cultures, where the sealed chambers are purged with a gas mixture of preset O_2_ concentration. Studies are conducted under the assumption that hypoxia remains unaltered throughout the 48 to 72 hour duration of such experiments. Since these chambers lack any sensor or detection system to monitor gas-phase O_2_, the cell-based data tend to be non-uniform due to the *ad hoc* nature of the experimental setup.

**Methodology:**

With the availability of low-cost open-source microcontroller-based electronic project kits, it is now possible for researchers to program these with easy-to-use software, link them to sensors, and place them in basic scientific apparatus to monitor and record experimental parameters. We report here the design and construction of a small-footprint kit for continuous measurement and recording of O_2_ concentration in modular hypoxia chambers. The low-cost assembly (US$135) consists of an Arduino-based microcontroller, data-logging freeware, and a factory pre-calibrated miniature O_2_ sensor. A small, intuitive software program was written by the authors to control the data input and output. The basic nature of the kit will enable any student in biology with minimal experience in hobby-electronics to assemble the system and edit the program parameters to suit individual experimental conditions.

**Results/Conclusions:**

We show the kit’s utility and stability of data output via a series of hypoxia experiments. The studies also demonstrated the critical need to monitor and adjust gas-phase O_2_ concentration during hypoxia-based experiments to prevent experimental errors or failure due to partial loss of hypoxia. Thus, incorporating the sensor-microcontroller module to a portable hypoxia chamber provides a researcher a capability that was previously available only to labs with access to sophisticated (and expensive) cell culture incubators.

## Introduction

Rapidly growing tumors, particularly those that are malignant, contain regions of low O_2_ within the tumor mass. These hypoxic regions help support the tumor against chemotherapy, radiation therapy, and alter the tumor's metabolism in such a way to help it invade and metastasize throughout the patient's body [1–6]. The studies suggest that an O_2_ concentration of 1.3% or below [oxygen partial pressure (pO_2_) of approximately ≤ 10 mm Hg] marks the threshold for altered metabolic changes in tumors; metabolism remains relatively unaltered at higher O_2_ concentrations [2]. Although it was previously thought that hypoxic regions are primarily located in the tumor “core”, recent investigations indicate that such hypoxic regions are more heterogeneously distributed within the bulk tumor [3, 7, 8].

Research laboratories routinely use portable modular incubator chambers (e.g. Billups-Rothenburg, Inc., CA, USA; StemCell Technologies, Inc., Vancouver, BC, Canada) to recapitulate the tumor hypoxic conditions during *in vitro* cell-culture studies [9]. These semi-spherical polycarbonate chambers of approximately 8 liters in volume cost approximately $700 for a modular chamber and a gas-flow meter. Tumor cell cultures are placed in the chamber and the chamber purged via a pair of ports for a predetermined time-period (e.g. 4 min at 20 L/min), usually with a pre-formulated gas mixture of 1% O_2_, 5% CO_2_, and 94% N_2_. The chamber is sealed and placed in a laboratory incubator (37°C) to culture the cells under hypoxic conditions. Manufacturers recommend a second purge with the gas pre-mix 1 hr later to remove any residual trapped air. However, since these modular units lack any sensor or detection system to monitor gas-phase O_2_, it is not possible to identify any changes to the O_2_ concentration in the chamber for the duration of the hypoxia experiment; the O_2_ level in the chamber is assumed to remain constant for the duration of the 48–72 hr experiment.

With the availability of numerous low-cost open-source (software and hardware that can be freely used, modified, and shared by anyone) microcontroller kits for educational and hobby electronics [10], a researcher can now interface these with environmental sensors (e.g. gas, temperature, pressure, humidity, or light detectors), and program the microcontroller to acquire data. The microcontroller programming steps are intuitive with user-friendly with instructions that do not require advanced programming skills. Thus, researchers in non-computer related fields could develop and embed these sensor-incorporated kits in basic scientific apparatus to monitor and record experimental parameters that were not previously possible.

In this report, we describe the development of a low-cost sensor module based on a popular microcontroller kit (Arduino Uno, Arduino.cc, Italy) for continuous measurement of O_2_ concentration in hypoxia chambers. Chamber O_2_ is monitored using a factory calibrated miniature gas sensor (Luminox-02, SST Sensing Ltd., Coatbridge, UK) that can simultaneously measure temperature and pressure also. The sensor is placed in the modular hypoxia chamber and connected to an externally located Arduino kit via hair-thin insulated copper wires (0.127 mm diameter, Temco Industrial Power, CA, USA), commonly used for winding electromagnets. The wires are routed through one of the gas-ports of the chamber, and thus do not require compromising the physical integrity of the chamber. The Arduino microcontroller is programmed with a simple instruction set (Arduino sketch) developed by the authors that acquires data from the O_2_ sensor at preset time intervals based on user input (for example, at 1 sec or 5 min intervals). Data are forwarded to a computer for the duration of the experiment via the microcontroller's USB port. The data (O_2_, temperature, pressure) are recorded and time-stamped using a free terminal program (CoolTerm, freeware.the-meiers.org), that can store up to 2 MB of serial data. The primary cost is for the O_2_ sensor ($75), while the microcontroller board and accessories cost $60.

Studies conducted by us using the above sensor- kit module indicated that continuous measurement of O_2_ is a necessity to maintain hypoxia (≤ 1% O_2_) in the modular chambers. Partial hypoxia (˃1% O_2_) can occur within minutes of purging the chamber, particularly with a gas mixture of 1% O_2_, 5% CO_2_, and 94% N_2_, the composition commonly used by many laboratories. Potential for likelihood of experimental failure due to partial hypoxia increases in proportion to the number of cell culture dishes or plates placed in the chamber at the beginning of an experiment. Our studies show that a second purge with gas, at least one hour after the first, is essential to maintain the required hypoxic conditions. We also show that a researcher can use the O_2_ sensor module described in this study to deliver and maintain a range of O_2_ concentrations between 0.1% and 1% in the hypoxic chamber via the use of a much less expensive anoxic gas mix of just CO_2_ and N_2_.

## Materials and Methods

### Summary

**Fig 1:**
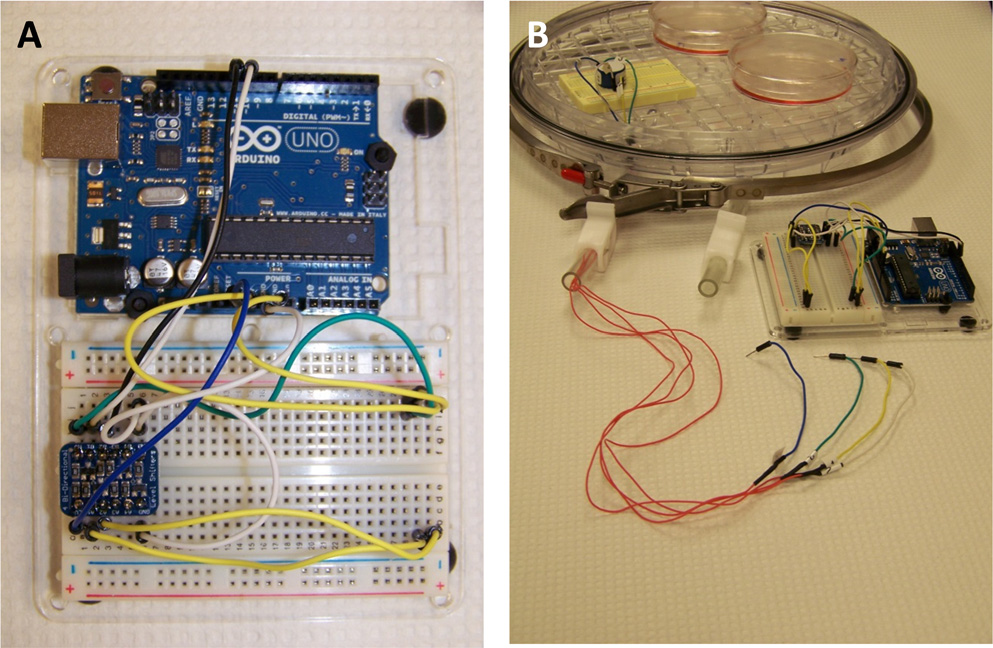
Oxygen sensor-hypoxia chamber module. (**A**) Close-up views of the Arduino Uno microcontroller board and half-sized breadboard mounted on acrylic plate. **(B)** Layout of the modular hypoxia chamber (with the oxygen sensor mounted on half-sized breadboard), the Arduino Uno microcontroller and the wire harness routed through one of the gas-flush tube ports, before assembly of the module (lid not shown). 30 AWG silicone-coated (orange colored) wires (cat. no. 2001, adafuit.com) were used in this photograph to clearly illustrate the wire harness.

The required electronic parts, their cost, and sources are listed in Table 1. The system is composed of an Arduino Uno (Revision 3) microcontroller board (6.8 x 5.3 cm), two half-sized "breadboards" (4.5 x 3.5 cm), one factory calibrated oxygen sensor (2 x 1.25 cm), and a 4-channel bi-directional logic converter. Breadboard jumper wires and an acrylic mounting plate (11 x 9 cm) were needed as accessories. The microcontroller board and one of the half-sized breadboards were affixed to the acrylic mounting plate for ease of handling and placed outside the hypoxia chamber (Fig 1A). The O_2_ sensor was mounted on the second half-sized breadboard and placed inside the hypoxia chamber (S1 Fig). The sensor was connected to the microcontroller using four insulated copper wires routed via one of the ports of the hypoxia chamber (Fig 1B). Thus, the entire assembly is modular, lightweight, detachable, and portable.

**Table 1:** Required electronic parts^a^ and accessories^b^.

Part	Cat. No.	Quantity	Source	Cost
Luminox-02 oxygen sensor	LOX-02	1	SST Sensing Ltd., Coatbridge, UK	$75.00
Arduino Uno Rev. 3 board	50	1	Adafruit.com	$25.00
Half-sized breadboard	64	2	Adafruit.com	$5.00
4-channel logic converter	757	1	Adafruit.com	$4.00
Temco 36 AWG wire spool	MW0542	1 (2 oz)	Amazon.com	$4.80
Acrylic mounting plate	275	1	Adafruit.com	$5.00
Heat shrink tubing (3/32")	55048407	1	RadioShack.com	$5.00
Breadboard jumper wires	153	1	Adafruit.com	$6.00

^a^ Parts can be obtained from many online electronics sources. The lowest cost sources are shown.

^b^ Other accessory items required: A 30 Watt or lower wattage (15 W) soldering iron equipped with a 15 W tip, 0.787 mm (0.032”) diameter flux-cored solder wire, and a heat-gun or hair dryer for “shrinking” heat-shrink tubing. Flexible tubing equivalent to peristaltic tubing (3.6 mm O.D. and 1.6 mm I.D.) or equivalent to pull the 36 AWG wire harness through the gas port of the hypoxia chamber.

### Microcontroller board

The Arduino Uno board (Arduino.cc, Italy) is an open source platform based on a low power 8-bit microcontroller (Atmega328; Atmel Corp., San Jose, CA) with 32K bytes in programmable flash memory. The board (16 MHz) has 14 digital I/O (Input/Output) pins, and 6 analog input pins. A USB port both powers the board and functions as a communication terminal for data transfer with a computer.

### Bi-directional logic converter

**Fig 2:**
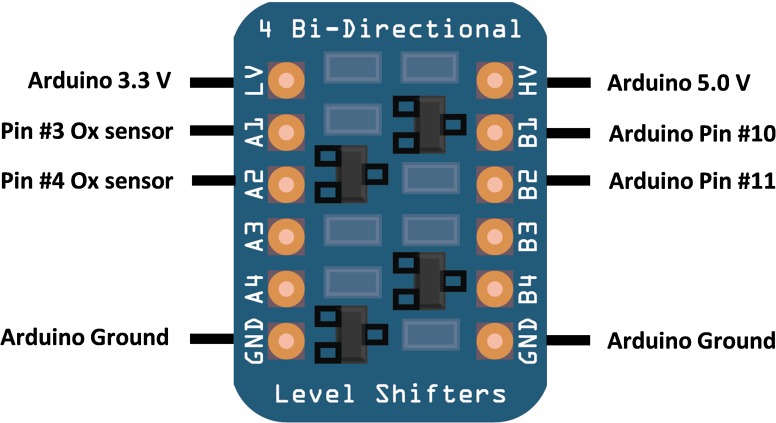
Schematic of the logic converter and connections. Pin designations of the 4-channel bi-directional logic converter (low-voltage pins A1, A2 and high voltage pins B1, B2 were used. A3, A4 and B3, B4 pins are extra, and not needed in this application). The logic converter is supplied as a mini printed circuit board (PCB) with a pair of 6-pin headers to facilitate mounting on a breadboard. We used a 30W soldering iron (RadioShack) fitted with a 15-watt soldering iron tip (cat. no. 64–2052, RadioShack) and 0.787 mm (0.032") flux-cored solder wire (cat. no. 64–005) to solder the PCB to the 6-pin headers. The soldered PCB was fitted on the half-sized breadboard mounted alongside the Arduino board.

The Arduino board communicates with sensors via 5.0 V I/O pins while the O_2_ sensor utilizes 3.3 V. Thus, a bi-directional logic converter (level shifter; BSS138, Fairchild Semiconductor) was needed to facilitate communication between the Arduino board and the O_2_ sensor. Pin assignments for wiring the oxygen sensor to logic converter are illustrated in Fig 2.

### Fluorescence based oxygen sensor

We used a luminescence-based oxygen sensor (oxygen optode) that operates on the principle of dynamic fluorescence quenching [11, 12]. The measurements can be based on a) the intensity of the fluorescence emission, where the intensity inversely correlates to the oxygen concentration, or b) the luminescence lifetime of the light emitted by the excited fluorophore where the measured oxygen concentration is inversely proportional to the luminescence lifetime. Sensors based on latter principle are more common, where a foil embedded with the fluorescent oxygen indicator (and exposed to test air) is excited with pulsed light in the "blue spectrum" (blue LED). The oxygen-quenched emitted light in the "red spectrum" is detected with a photodiode. Relationship between O_2_ concentration and the decay time is described by the Stern-Volmer equation for O_2_:

$$\left[O_{2}\right]=\frac{1}{K_{sv}}\;\left(\frac{\tau_{o}}{\tau_{f}}-1\right)$$

where K_*SV*_ is the Stern-Volmer quenching constant; *τ*_*o*_ the fluorescence lifetime in the absence of O_2_; *τ*_*f*_ the fluorescence lifetime in the presence of O_2_.

Per manufacturer's literature (LOX-02, SST Sensing Ltd., Coatbridge, UK), the O_2_ sensor we used in our studies is a low-power, factory-calibrated RoHS compliant device that is both pressure and temperature compensated. The sensor has a >5 year lifetime and a <30 mSec response time. O_2_ is measured in the range of 0 to 25% (0 to 300 mbar). The sensor can operate at high relative humidity (0 to 99% non-condensing), an important parameter to consider when placed inside a sealed humidified hypoxia chamber, and has an operating temperature range of up to 60°C. While the temperature output from the sensor is accurate to 0.1°C, it provides relative temperature data.

**Fig 3:**
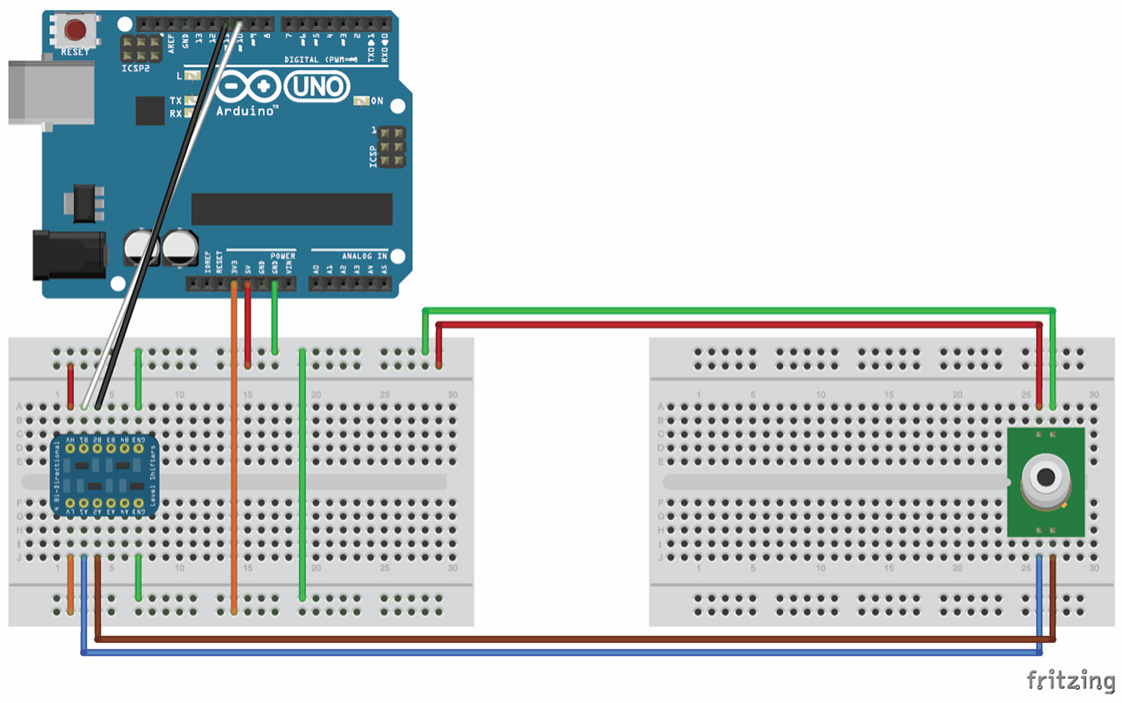
Outline of the wiring diagram. A Fritzing (fritzing.org) sketch is presented outlining the wiring connections between the microcontroller, the logic converter, and the oxygen sensor.

The sensor is powered at 5 V (7.5 mA; 20 mA peak). Serial communication is via a 3.3 V RS232 communication protocol (datasheet available at sstsensing.com/product/luminox-optical-oxygen-sensor). Four non-symmetrically oriented pins mount the sensor to the breadboard (pin 1, 5V; pin 2, 0V; pin 3, 3.3V sensor transmit-TX; pin 4, 3.3V sensor receive-RX). The entire wiring circuit is illustrated as a “Fritzing” diagram (Fritzing.org) (Fig 3). The sensor sends O_2_, temperature and pressure data at 1 sec intervals to the Arduino board. Assembly and routing of the wires that transmit data from the sensor to the Arduino board are described in S2 Fig.

### Hypoxia Experiments

We used a modular incubator chamber (MIC-101; Billups-Rothenberg, Inc., Del Mar, CA) for our experiments. A 10 cm open tissue culture dish containing 10 ml H_2_O was placed on the bottom of chamber to maintain humidity. Most of the described experiments involved the use of two 10 cm diameter tissue culture plates (Corning) each containing 1 x 10^6^ brain tumor cells [U87MG or U251MG, American Type Culture Collection (ATCC), Manassas, VA] in 10 ml RPMI-media supplemented with 10% fetal bovine serum (FBS). One set of experiments involved the use of a 24-well tissue culture plate with each well containing 5 x 10^4^ cells in 1 ml of same media. The oxygen sensor mounted on one of the half-sized breadboards was placed inside the chamber. The four power/data wires (routed into the chamber as described in S2 Fig) were connected to the respective pins of the oxygen sensor. The external termini of the wires were connected to the corresponding pins on the breadboard mounted alongside the Arduino board (Fig 3). The chamber was closed and purged with a 95% N_2_, 5% CO_2_ anoxic gas mixture (the gas-exchange port free of data transfer wires was used as the intake port; the port used for data transfer wires was used as the exhaust port). The chamber was purged for 4 min at 20 L/min per manufacturer’s instructions. A flow meter (SFM3001; 3–25 LPM adjustable flow rate) from the same manufacture was used to regulate the gas flow. During initial experiments, the accuracy of the O_2_ sensor was validated via the use of a clinical grade in-line O_2_ analyzer (OM-25AE MaxO_2_; Maxtec, Inc., Salt Lake City, UT) that was attached to the exhaust port of the hypoxia chamber.

Both gas exchange ports were clamped immediately after the purge procedure. The assembly (hypoxia chamber attached to the Arduino board) was placed on the upper shelf of a 37°C incubator (we used a Forma Scientific Model 3110 tissue culture incubator). The Arduino board was connected to a laptop computer placed outside the incubator via a 6 foot USB cable that was routed through the rear gas exhaust port of the incubator. The computer (which can be Windows XP or Windows 7 based) had Arduino software (IDE 1.6.1), the CoolTerm terminal program, and the program for hypoxia measurement (Arduino sketch, see next) preinstalled. Note: The Arduino software and CoolTerm programs are available for Apple and Linux-based computers also.

### Arduino sketch for O_2_, temperature, and pressure data acquisition

The Arduino microcontroller can be programmed to collect data output from a sensor as string arrays [13–15]. Arduino IDE software (version 1.6.1, arduino.cc/en/Main/Software) was used to program the microcontroller via its USB port. We used public domain code available for Arduino (arduino.cc/en/) to generate the sketch (S1 Text). The program was written to acquire data at 5 min intervals during a 48 to 72 hr hypoxia based experiment, with the option to collect data at 1 sec intervals when the chamber is being purged with the gas-mixture. A user can switch between the intervals via a simple text based command through the CoolTerm terminal program. The program settings for CoolTerm (ver. 1.4.6 b1, build 248 or later) are described in S1 Table. Stepwise instructions for initiating data collection are described in S2 Table.

**Fig 4:**
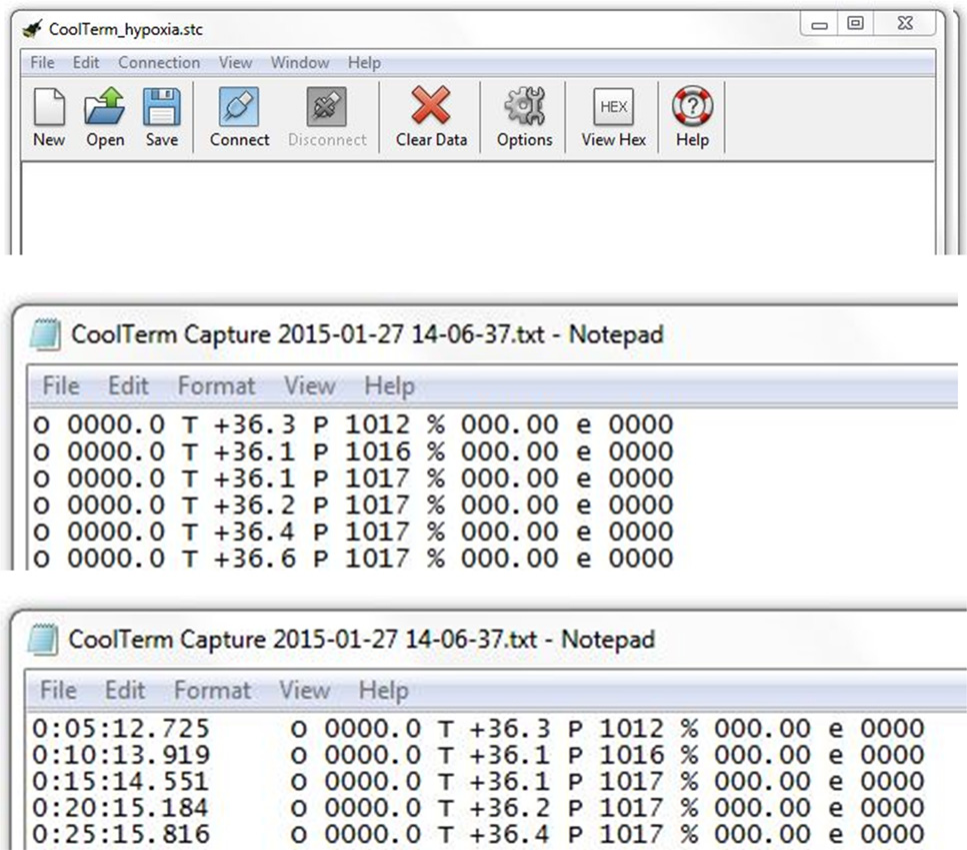
Screen-capture images of CoolTerm terminal window. **Upper panel**: CoolTerm quick access bar and ribbon; **middle panel**: format of the initial data output from CoolTerm; **lower panel**: format of the final data saved by CoolTerm with time-stamps (saved and opened as a Microsoft Notepad file).

Data from the current study were analyzed with Microsoft Excel or SigmaPlot software (Systat Software, San Jose, CA) and plotted to illustrate the changes in O_2_ concentration. In brief, Time, Temperature, Partial pressure and O_2_% data columns were imported from the saved CoolTerm text files (Fig 4) into the program of choice using Tab, Space and Colon delimited settings. Selected data columns were plotted as 2D charts. Note: These steps are unnecessary if a researcher simply needs to monitor O_2_ levels—which can be directly read from the CoolTerm window, or via the Arduino IDE program itself.

### Readjustment of O_2_% mid-experiment

When the O_2_ level in the hypoxia chamber increases above a threshold level during an experiment (for example above 1%), the chamber can be re-purged at a slow rate to return the O_2_% to required hypoxic levels. Stepwise instructions for this procedure are provided in S2 Table.

### Use of an anoxic gas mixture to adjust chamber O_2_%

Laboratories routinely use a 1% O_2_ gas pre-mix to purge the hypoxia chambers. In addition to the potential for experimental failure due to partial hypoxia (> 1%) when O_2_ is out-gassed by the cell culture media, use of a gas pre-mix precludes researchers from testing a range of O_2_ concentrations in their studies. However, with the introduction of a sensor into the hypoxia chamber, it becomes possible to use an anoxic gas-mix to conduct programmed purging of the chamber and adjust the O_2_ tension to any desired level. This facilitates greater experimental flexibility.

Programmed adjustment of chamber O_2_% can be accomplished as follows; the closed modular chamber containing the cell cultures, and the Arduino board are first placed in the 37°C incubator without an initial 4 min, 20 L/min gas purge. The Arduino board is connected to the computer to collect data at 1 sec intervals as described in S2 Table. Then, the anoxic gas mixture is purged through the modular chamber at 20 L/min until O_2_% drops from the initial 21% to 5%. The flow rate is then reduced to 5 L/min and the O_2_ level allowed to drop to the desired level (e.g. 0.5%) and the ports clamped closed to seal the chamber. These steps can be completed within 5 min, which minimizes the duration to which the incubator door needs to be kept partially open. Afterwards, the CoolTerm program is reset to read at 5 min intervals (S2 Table). Thus, the chamber O_2_% can be preset to a desired O_2_ tension between 0.1–1%, the hypoxic settings routinely used for most hypoxia experiments.

## Results

The system we assembled using "off-the-shelf" low-cost electronic components was capable of continuous monitoring of O_2_ levels in hypoxia related cell-culture experiments for over a time span of 72 hours or longer. An added benefit of the sensor was its ability to measure partial pressures and temperature at each time point.

### A single purge of the modular chamber with a hypoxic gas-mix is insufficient to maintain long-term hypoxia

**Fig 5:**
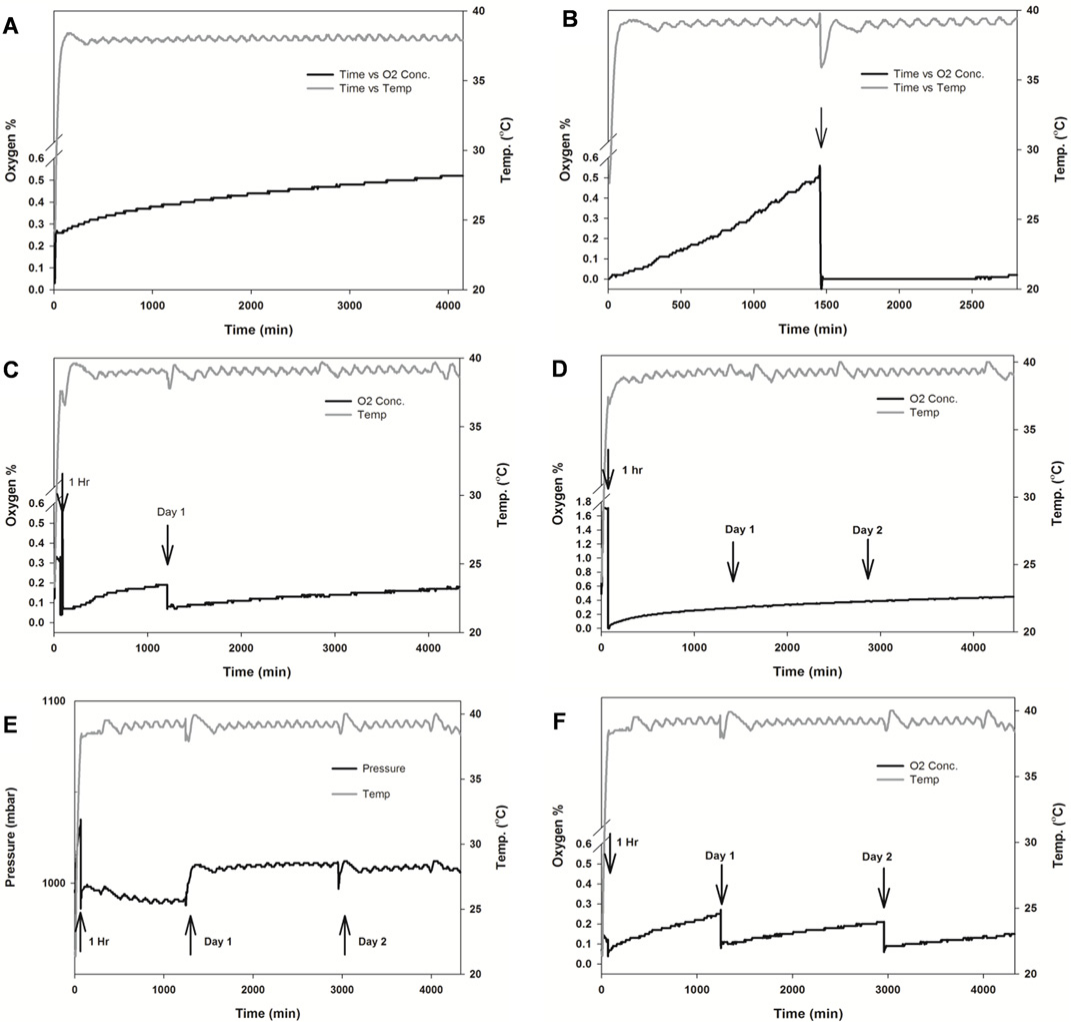
Representative oxygen and temperature data transmitted by the sensor under experimental conditions. (**A**) change in chamber O_2_% upon a single 4 min (20 L/min) anoxic gas purge conducted in the presence of cell cultures. (**B**) lack of a rapid initial increase in O_2_% when chamber is devoid of any cell cultures, liquid media or water. (**C**) change in chamber O_2_% with a post-1 day gas purge (in the presence of cell cultures). (**D**) change in chamber O_2_% with daily gas purges in the presence of cell cultures. (**E**) pressure profile of experiment listed in **D**. (**F**) change in chamber O_2_% profile upon inclusion of ten cell culture plates.

We first tested the ability to achieve hypoxia (≤ 1% O_2_) with a single purge of the hypoxia chamber with the anoxic gas mixture (20 L/min, 4 min). Although this reduced the chamber O_2_% approximately to 0.05%, the O_2_% recovered within minutes. We observed on average (n = 3) a 0.3% ± 0.1% increase in O_2_ in the chamber within the first 5–10 minutes. Subsequent rise in O_2_ in the chamber was slow (0.003% hr^-1^) most likely due to slower equilibration with dissolved oxygen in tissue culture media (2 x 10 ml) and H_2_O (10 ml). O_2_ increased to approximately 0.5% during the next 72 hrs (Fig 5A). Thus, a single-step purge with a 1% O_2_ gas-mixture (routinely used by researchers in hypoxia studies) would have created partial hypoxia conditions in the chamber within the first hour.

### Increase in O_2_% in the hypoxia chamber is due to outgassing by tissue culture vessels

We verified that the initial increase in chamber O_2_ was due to rapid displacement of O_2_ from the headspace of the covered tissue culture plates and by liquid media in culture vessels to the chamber air by testing the empty chamber with a single (20 L / min, 4 min) gas-purge. A rapid recovery in O_2_% was not observed. It took 24 hrs for the chamber O_2_ to increase to 0.5% (Fig 5B). After the chamber was re-purged at this point, O_2_% did not show any long term increase. The data indicated that the increase in O_2_ observed in the previous experiment was due to O_2_ out-gassing from headspace of cell culture vessels and from media and H_2_O placed in the vessels.

### A post-one hour gas-purge of the modular chamber is essential to maintain long-term hypoxia

Hypoxia chamber manufacturers recommend, but do not require, a second gas-purge one hour after the first. As described in Fig 5A, chamber O_2_ reached 0.3% within the first hour after a single initial gas-purge (with cell culture vessels are included in the chamber). However, upon adding a second (post-1 hr) gas purge O_2_% was maintained below the hypoxia threshold of 0.5% for the next 24 hrs (Fig 5C). Although O_2_% slowly increased, the chamber maintained hypoxia long term (72 hrs). We then tested the increase in O_2_ levels when the chamber was purged every 24 hrs (Fig 5D) where the O_2_ level was brought back down to <0.1% at each step. However, we could not see a significant control of O_2_ with these additional steps in comparison to data from Fig 5C, indicating that a single post-1 hr gas purge was sufficient to maintain long-term hypoxia.

Long-term slow increase of O_2_ in the chamber could also occur due to potential leakage of air across the O-ring or the clamped gas-exchange port tubing. We eliminated this possibility by monitoring pressure data from the O_2_ sensor (Fig 5E), which indicated maintenance of a positive pressure gradient throughout the 72 hr duration of the above experiment. The slight increase in chamber pressure occurs when the sealed chamber is placed in the tissue culture incubator, causing the chamber temperature to increase from 25°C (room temperature) to 37°C. Thus, the pressure data verified the integrity of the chamber seal and gas exchange tubing clamps.

### A second gas-purge is critical to maintain long-term hypoxia when a large number of tissue culture vessels are placed in the hypoxia chamber

Researchers frequently place a large number of tissue culture plates in these hypoxia chambers particularly when a series of cell-cultures are to be tested for their biological responses under hypoxia. We simulated the conditions by placing five plates each of the glioma cell cultures (for a total number of 10 plates) in the chamber and purged the closed chamber with the anoxic gas mix for 4 min (20 L/min). We observed an increase in O_2_ to 1.7 ± 0.3% within the first hour, clearly indicating rapid induction of non-hypoxic conditions. A second (post 1-hr) gas-purge was completed and the O_2_ level monitored for the next 72 hrs, which indicated the maintenance of O_2_% below the 1% hypoxia threshold (Fig 5F). The results indicate the critical need to monitor chamber O_2_ level when a large number of cell cultures are placed in these modular chambers. Had a single purge with a gas mixture of 1% O_2_ been used (as is routine in many laboratories), the final O_2_ level would have exceeded standard maximum experimental hypoxic level of 1% O_2_ within 24 hrs, with a compromised outcome for the studies.

### Programmed purging of the hypoxia chamber with an anoxic gas-mix can be used to adjust O_2_% to any desired experimental level

**Fig 6:**
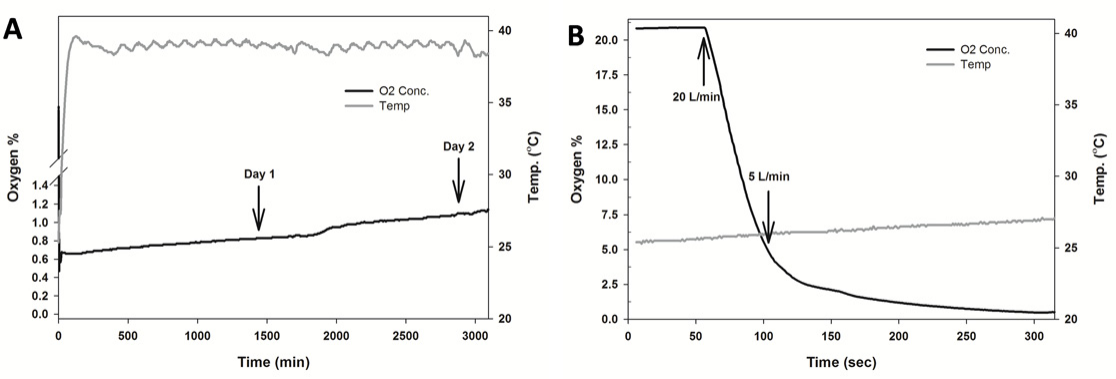
Oxygen and temperature changes during programmed adjustment of hypoxia. A single 24-well plate of cell cultures was used and monitored over 48 hrs. (**A**) The sealed modular hypoxia chamber was placed in cell culture incubator (without a purge with anoxic gas-mix). The chamber was then flushed with the anoxic gas-mix at an initial flow rate of 20 L/min (approx. 2 min) to lower the O_2_ level from 21% to 5%. This was followed by a slower flow rate of 5 L/min (approx. 3 min) until O_2_% reached 0.5%. (**B**) expanded view of the change in O_2_% during the initial 5 min programmed purge procedure.

With the ability to continuously monitor chamber hypoxia, a researcher should be able to induce a range of hypoxic levels via the use of an anoxic gas mix. We tested this possibility using a 24-well plate format, the commonly used system for hypoxia based reporter gene assays [16]. The chamber was purged with the anoxic-gas mix with the O_2_ sensor set to read at 1 sec intervals as described in S2 Table. Chamber O_2_% was monitored until O_2_ level dropped to 0.5% (Fig 6A and 6B) and the gas-exchange ports sealed (the 0.5% O_2_ level was selected to reflect the lower O_2_ threshold used by some investigators during hypoxia based cell-culture experiments). Data collection rate was reset to 5 min intervals and the O_2_ level monitored for 48 hrs, the usual duration for hypoxia based reporter assays. We observed that the system could maintain O_2_ level at or below 1% with this procedure, without further adjustments. Thus, a researcher could use an anoxic gas mix and a similar programmed gas-purge protocol to maintain hypoxia at a range of predetermined O_2_ levels.

### The O_2_ sensor can provide accurate yet relative temperature data

The O_2_ sensor is capable of providing relative temperature data. In our hands, under standard laboratory atmosphere and temperature (22°C) the sensor gave a reading that was 2.3°C higher (24.3°C). In a humidified cell-culture incubator (5% CO_2_, 16% O_2_, and 79% N_2_ atmosphere and 37°C), the reading was 4.2°C higher (41.2°C). Thus, if direct temperature readouts are required during an experiment, a prior evaluation of the readout from the sensor vs. the actual incubator temperature will be necessary. In all instances of collected data, temperature oscillations were observed (Figs 4 and 5) that were parallel to the thermostat-based temperature controls of the cell-culture incubator, indicating the highly sensitive nature of the temperature probe embedded within the oxygen sensor.

## Discussion

We describe a low-cost portable oxygen sensor platform that can be easily assembled by researchers as a useful analytical tool to monitor and log oxygen tension during hypoxia based cell-culture studies. While the experiments reported here are on mammalian cells and culture conditions, the sensor module can be easily applied to monitor experiments that involve other eukaryotes such as plant or yeast based cell cultures. Numerous Arduino microcontroller-based prototypes that may be useful to the biomedical research community for further development have been reported (medicarduino.net) including monitors for EEG, ECG, pulse oximeters, heart-rate monitors, and breathalyzers. Although these are primarily educational electronics based projects, they provide a first stage prototype for those in the research community interested in incorporating or developing sensor- or remote- monitoring systems in support of their research [17].

While sophisticated incubator chambers equipped with their own temperature, pressure, humidity and pO_2_ regulation are available [18, 19], they are not economically feasible for most cell-culture laboratories due to the high cost of purchase (approximately $25,000) and maintenance. Thus, the modular chambers described in our study have become the apparatus mostly used for hypoxia based *in vitro* cell-culture research.

The factory-calibrated O_2_ sensor incorporated into our Arduino module provides an accurate tool for continuous monitoring of intra-chamber oxygen tension throughout a 2–3 day hypoxia experiment. The high sensitivity of the sensor and the stability of the measurement will enable researchers to obtain real-time information of the hypoxia conditions, and readjust O_2_ levels if necessary. Our data showed that even an initial 4 minute (20 L/min) purge of the hypoxia chamber with an anoxic gas mixture will give rise to an O_2_ concentration of approximately 0.5% within 24 hrs. Thus, experimental variables can give rise to partially hypoxic conditions, particularly when most investigators purge these chambers with a gas composed of 1% O_2_, or, if a significant number of cell culture dishes are enclosed in the hypoxia chamber. A second purge with gas (4 min, 20 L/min) one hour after the first, is essential to maintain the required hypoxic conditions, despite being only a recommendation by the manufacturers of modular hypoxia chambers. We also show that a researcher can use a much less expensive anoxic gas mix to deliver a range of O_2_ partial pressures to the hypoxic chamber while monitoring the chamber gas phase with the O_2_ sensor. Thus, the sensor platform provides a low-cost setup to accommodate a range of O_2_ concentrations for use, rather than forcing researchers to rely on one pre-determined O_2_ concentration for their hypoxia studies.

In summary, we describe a small-footprint, low-cost monitor for continuous recording of oxygen tension in modular chambers widely used by research laboratories undertaking hypoxia-based studies. An oxygen sensor, an Arduino microcontroller, a Windows, Linux or Apple-based laptop or desktop computer running a freeware terminal program for data logging are the primary required components.

## Supporting Information

S1 Fig Photograph of oxygen sensor mounted on half-sized breadboard inside the modular hypoxia chamber. (DOCX)

S2 Fig Data transfer wire assembly. (DOCX)

S1 Table Program settings for CoolTerm for serial communication with the Arduino board. (DOCX)

S2 Table Stepwise instructions for initiating data collection in CoolTerm program. (DOCX)

S1 Text The Arduino Sketch for data transfer and serial communication. (DOC)

## References

[1] Hockel M, Vaupel P. Biological consequences of tumor hypoxia. Seminars in oncology. 2001;28(2 Suppl 8):36–41. . 11395851

[2] Hockel M, Vaupel P. Tumor hypoxia: definitions and current clinical, biologic, and molecular aspects. Journal of the National Cancer Institute. 2001;93(4):266–76. . 11181773 10.1093/jnci/93.4.266

[3] Vaupel P, Mayer A. Hypoxia in cancer: significance and impact on clinical outcome. Cancer metastasis reviews. 2007;26(2):225–39. 10.1007/s10555-007-9055-1 . 17440684

[4] Wilson WR, Hay MP. Targeting hypoxia in cancer therapy. Nature reviews Cancer. 2011;11(6):393–410. 10.1038/nrc3064 . 21606941

[5] Colen CB, Shen Y, Ghoddoussi F, Yu P, Francis TB, Koch BJ, et al Metabolic Targeting of Lactate Efflux by Malignant Glioma Inhibits Invasiveness and Induces Necrosis: An In Vivo Study. 2011 10.1593/neo.11134 21750656 PMC3132848

[6] Mathupala SP, Colen CB, Parajuli P, Sloan AE. Lactate and malignant tumors: a therapeutic target at the end stage of glycolysis. Journal of bioenergetics and biomembranes. 2007;39(1):73–7. Epub 2007/03/14. 10.1007/s10863-006-9062-x 17354062 PMC3385854

[7] Evans SM, Judy KD, Dunphy I, Jenkins WT, Hwang WT, Nelson PT, et al Hypoxia is important in the biology and aggression of human glial brain tumors. Clinical cancer research: an official journal of the American Association for Cancer Research. 2004;10(24):8177–84. 10.1158/1078-0432.CCR-04-1081 . 15623592

[8] Evans SM, Judy KD, Dunphy I, Jenkins WT, Nelson PT, Collins R, et al Comparative measurements of hypoxia in human brain tumors using needle electrodes and EF5 binding. Cancer research. 2004;64(5):1886–92. . 14996753 10.1158/0008-5472.can-03-2424

[9] Wu D, Yotnda P. Induction and testing of hypoxia in cell culture. Journal of visualized experiments: JoVE. 2011;(54). 10.3791/2899 21860378 PMC3217626

[10] Allan A. Arduino Uno vs BeagleBone vs Raspberry Pi 2013. Available: https://makezine.com/2013/04/15/arduino-uno-vs-beaglebone-vs-raspberry-pi/.

[11] Opitz N, Lubbers DW. Theory and development of fluorescence-based optochemical oxygen sensors: oxygen optodes. International anesthesiology clinics. 1987;25(3):177–97. . 3323063 10.1097/00004311-198702530-00011

[12] Lubbers DW. Optical sensors for clinical monitoring. Acta anaesthesiologica Scandinavica Supplementum. 1995;104:37–54. . 7660749 10.1111/j.1399-6576.1995.tb04254.x

[13] Monk S. Programming Arduino: getting started with sketches New York: McGraw-Hill; 2012 xiv, 162 p. p.

[14] Margolis M. Arduino cookbook 2nd ed. Sebastopol, Calif.: O'Reilly; 2012 xx, 699 p. p.

[15] Scherz P, Monk S. Practical electronics for inventors Third edition ed. New York: McGraw-Hill; 2013 xxv, 1014 pages p.

[16] Bronstein I, Fortin J, Stanley PE, Stewart GS, Kricka LJ. Chemiluminescent and bioluminescent reporter gene assays. Anal Biochem. 1994;219(2):169–81. 10.1006/abio.1994.1254 . 8080073

[17] Li M. Arduino in research and biotech 2015. Blog]. Available: http://blog.atmel.com/2015/02/24/arduino-in-research-and-biotech/.

[18] Bates MK. Culturing Cells Under Hypoxic Conditions for Biologically Relevant Results. American laboratory 2012.

[19] Rau M. True Hypoxia Replication: Creating Optimal Conditions for Cell-Based Research. American laboratory 2014.

