# Supplementary figures and images for: A Lab Assembled Microcontroller-Based Sensor Module for Continuous Oxygen Measurement in Portable Hypoxia Chambers
Source: PLoS One. 2016 Feb 10;11(2):e0148923. doi: 10.1371/journal.pone.0148923 (PMC4749204; doi:10.1371/journal.pone.0148923)

**S1 Fig.**


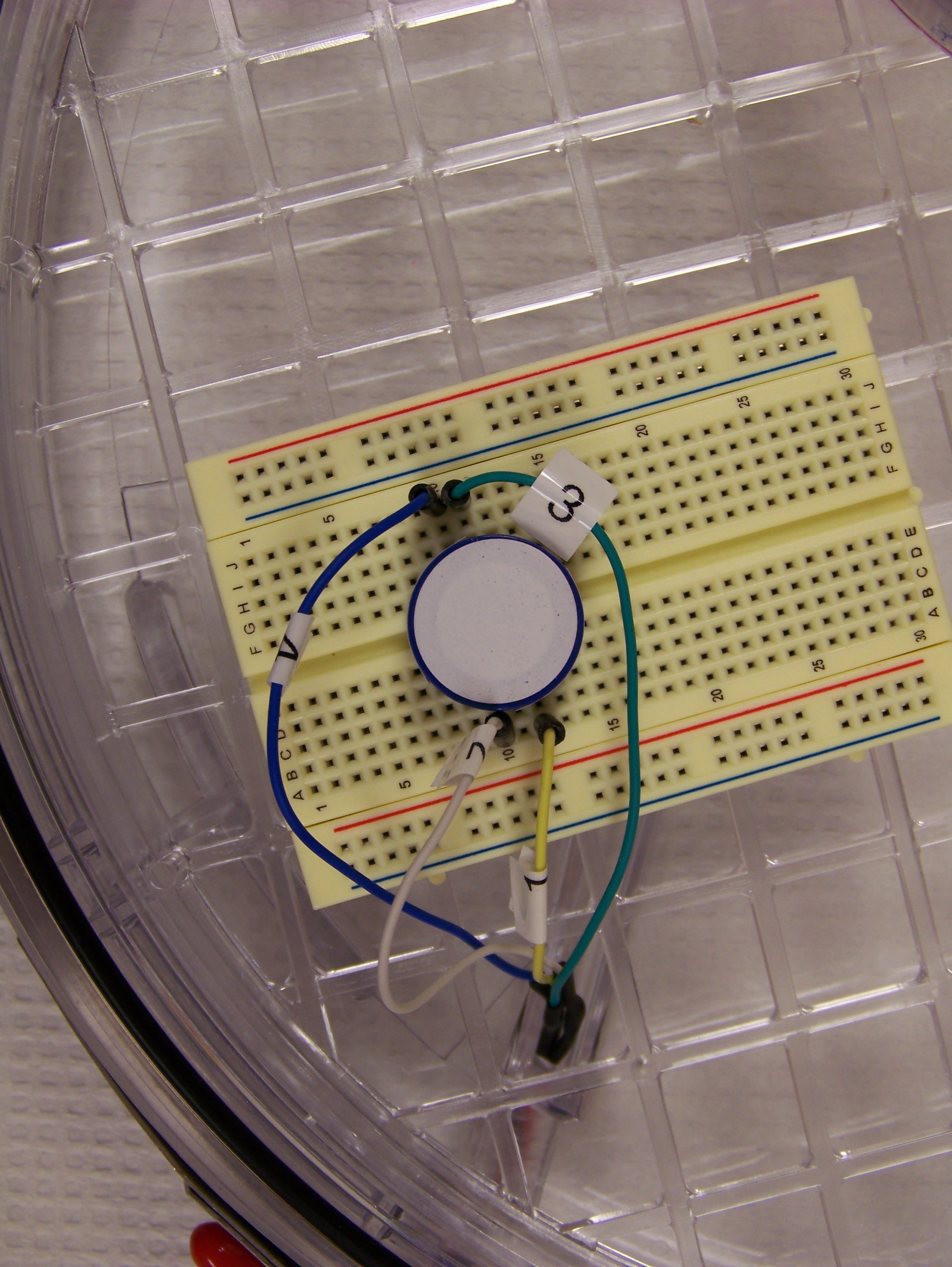

Supplement: S1 Fig — (DOCX) [file pone.0148923.s001.docx]
