# Supplementary material for: A Lab Assembled Microcontroller-Based Sensor Module for Continuous Oxygen Measurement in Portable Hypoxia Chambers
Source: PLoS One. 2016 Feb 10;11(2):e0148923. doi: 10.1371/journal.pone.0148923 (PMC4749204; doi:10.1371/journal.pone.0148923)

**S2 Fig. Data transfer wire assembly.**

(**A**) **Photograph of wire assembly with individually colored breadboard wires soldered to 36 AWG insulated copper wires for communication between the oxygen sensor and the Arduino board.** The ruler on the right identifies the scale in cm. Four alternate colored breadboard wires (25 cm) (part no. 153, Adafruit.com) were cut in half, and each colored pair soldered to 50 cm lengths of insulated 36 AWG (0.005"; 0.127 mm diameter) copper wires (cat. no. MW0542, Soderon 155; Temco Industrial Power Supply, Fremont, CA). 2 cm long, 3/32" diameter, heat-shrink tubing (cat. no. 55048407; RadioShack) sections were slipped over the breadboard wire halves, and 5 mm sections of insulation stripped off cut-ends of the breadboard wires. The 36 AWG copper wires were simply wrapped around the exposed termini and soldered (Soderon wires do not need removal of insulation prior to soldering). Then, heat-shrink tubing sections were positioned over the soldered connections and heat-sealed for insulation.


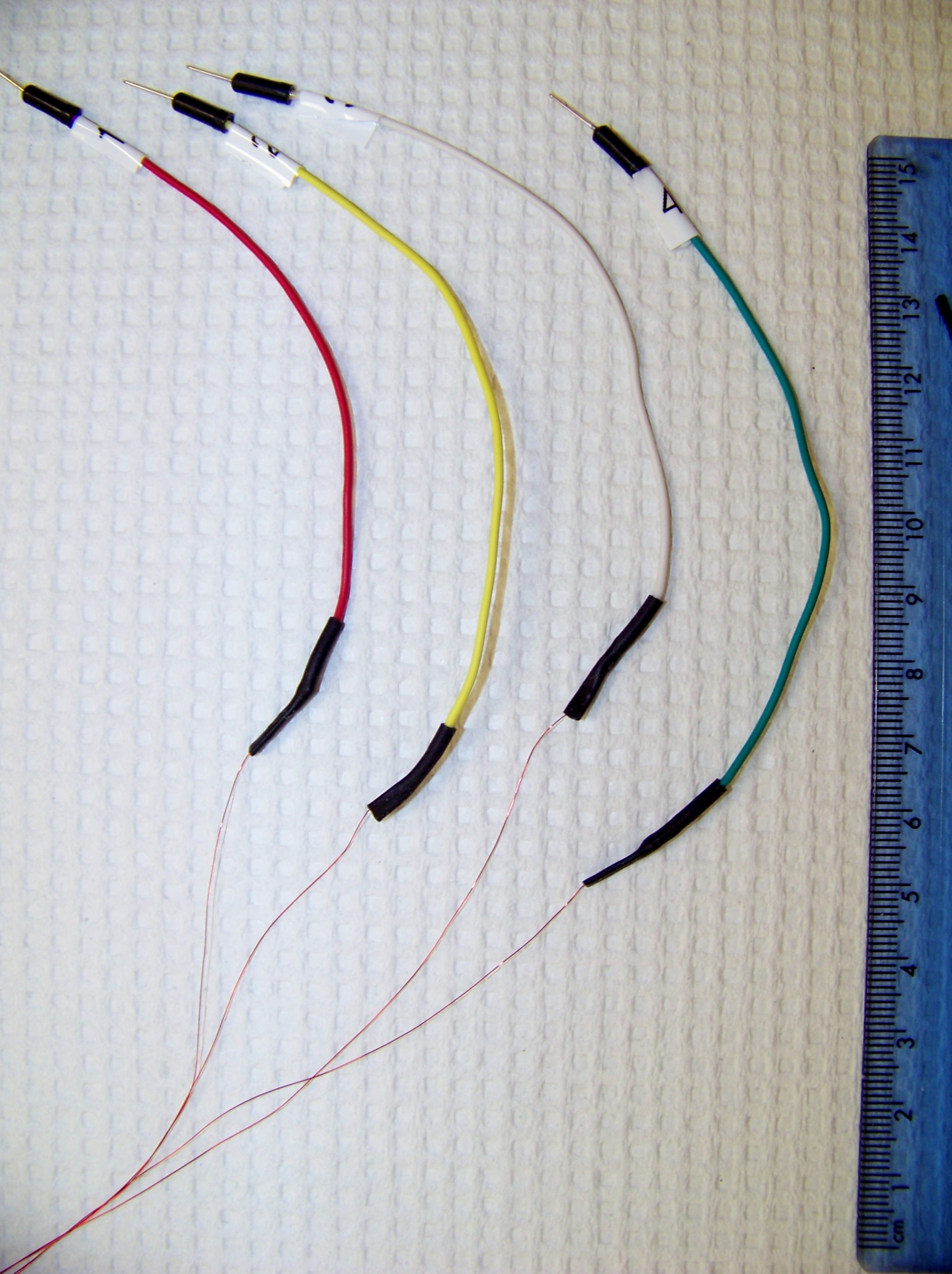


**The following steps describe the method we used to run the wire assembly through one of the gas exchange ports of the hypoxia chamber.** (**B**) Plastic clamp on one of the ports was removed and a 20 cm long section of flexible peristaltic pump tube (3.6 mm OD x 1.6 mm ID; we used Bio-Rad cat. no. 7319007) or similar routed through the flexible Tygon tube of the port.


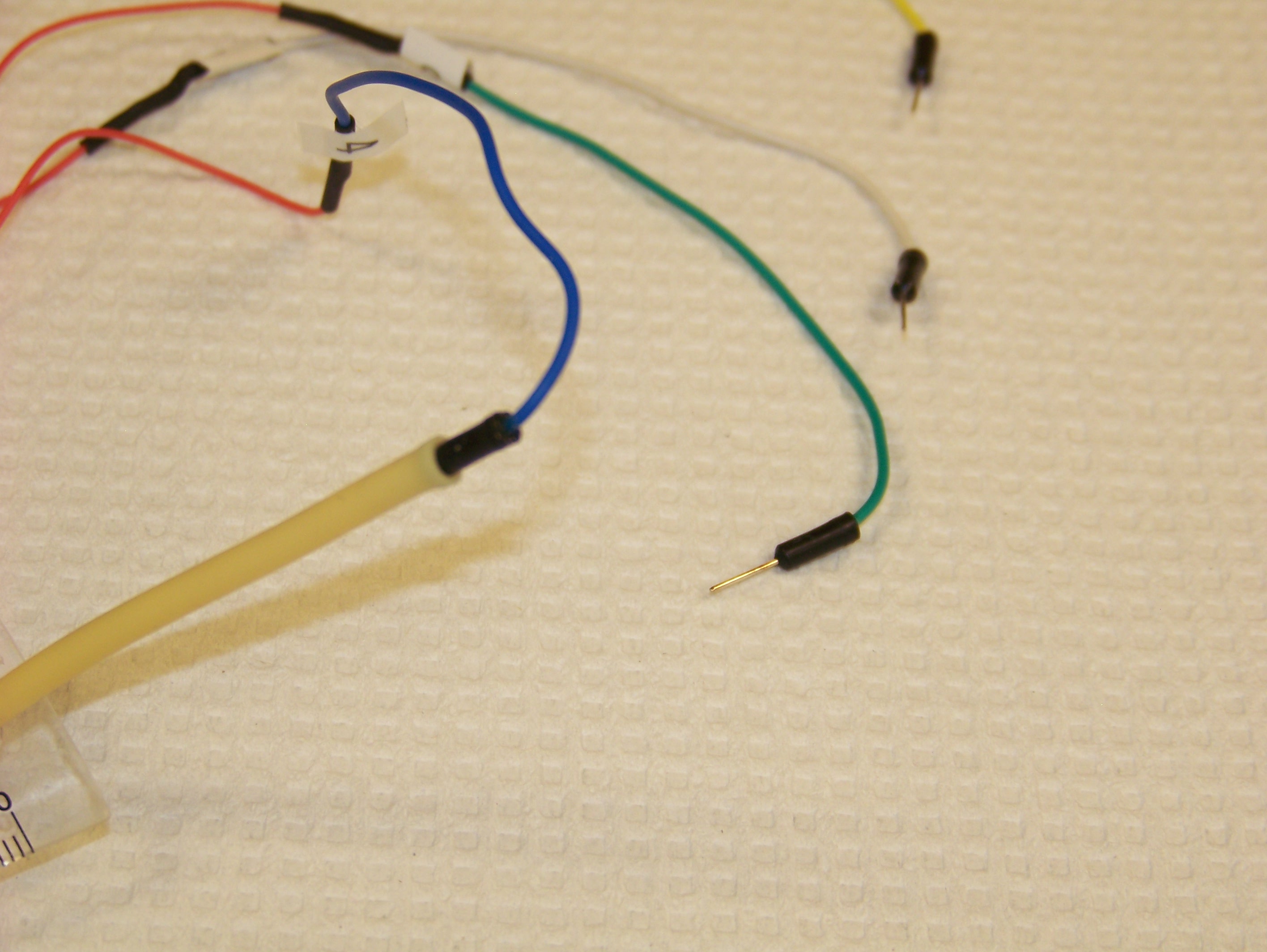


(**C**) Terminal of one of the assembled breadboard wires was inserted into the peristaltic tube-end, and the peristaltic tube pulled back through the Tygon tubing . The process was repeated until all four wires were run through the port. Then, the plastic clamp was repositioned over the Tygon tube.


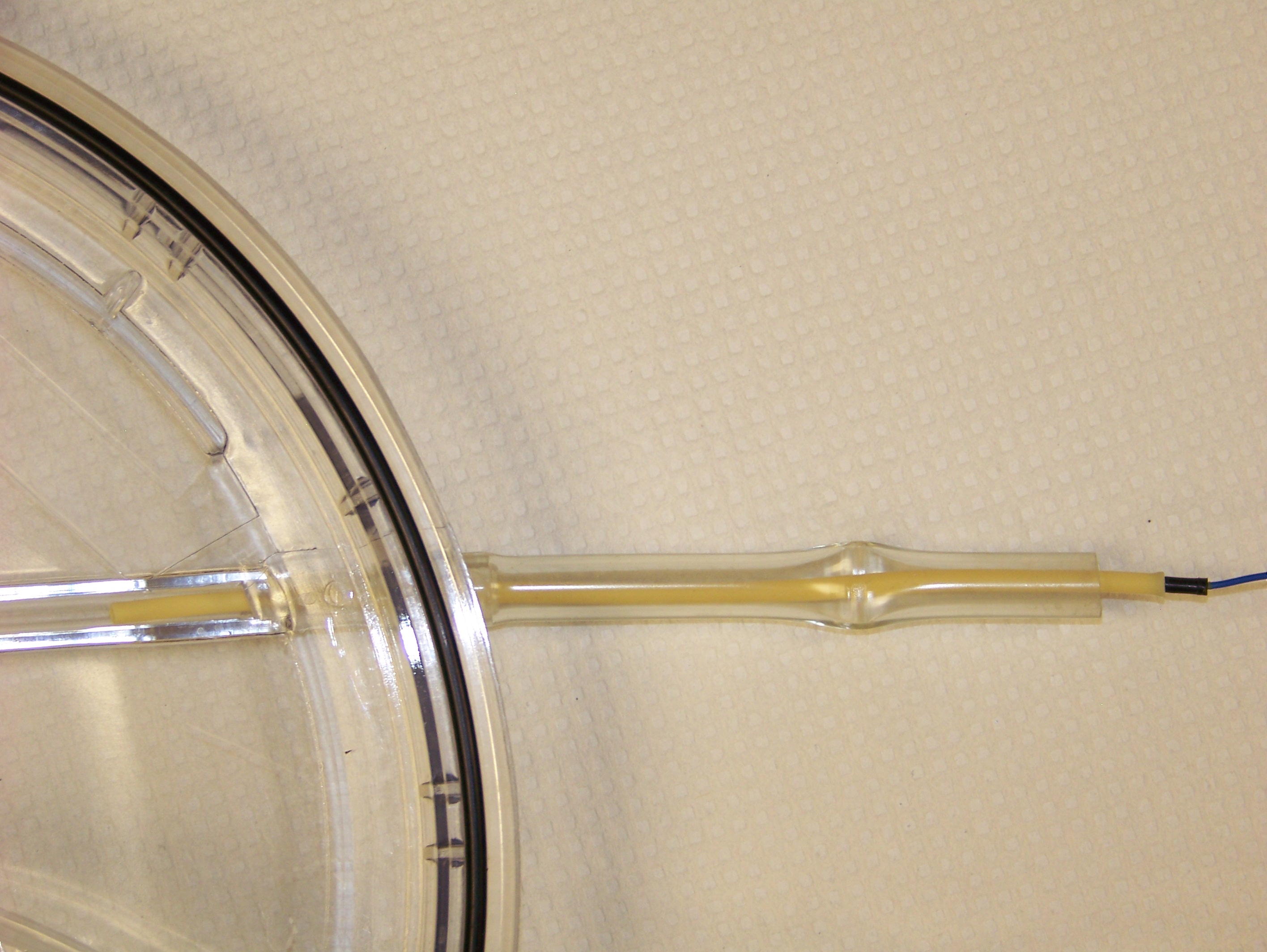

Supplement: S2 Fig — (DOCX) [file pone.0148923.s002.docx]
