# Supplementary material for: A Lab Assembled Microcontroller-Based Sensor Module for Continuous Oxygen Measurement in Portable Hypoxia Chambers
Source: PLoS One. 2016 Feb 10;11(2):e0148923. doi: 10.1371/journal.pone.0148923 (PMC4749204; doi:10.1371/journal.pone.0148923)
Supplement: S1 Table — (DOCX) [file pone.0148923.s003.docx]

**S1 Table. Program settings for CoolTerm^©^ for serial communication with the Arduino board.**

| Get to the program settings via **Connection ⮱ Options** |
| --- |
| ***Serial port*:** Port 1 or higher (depends on the laptop computer's USB port setting); Baud rate = 9600; Data bits = 8; Parity = none; Stop bits = 1; DTR on; RTS on. (Leave other boxes blank)  ***Terminal*:** Terminal mode = Raw data; Enter key emulation = CR+LF; Custom seq. (hex) = 00 1B; Special character handling: ✓convert non-printable characters. (Leave other boxes blank)  ***Receive*:** Receive buffer size = 200,000; Capture format = raw data; ✓Add time stamps to received data; ✓wait for termination string; Termination string = 0D 0A; Type = "relative time"; ✓leave file open while capturing. (Leave other boxes blank)  ***Transmit*:** Leave all boxes blank. Do not select any setting.  ***Miscellaneous*:** Leave all boxes blank. Do not select any setting. |

***Note:** Save the new program settings with a new file name such as "CoolTerm_Hypoxia". The default name "CoolTerm_0" is reserved for default settings of the program.

^©^CoolTerm Version 1.4.5 (Build 242)

Author: Roger Meier ([roger.meier@pobox.com](mailto:roger.meier@pobox.com))

<http://freeware.the-meiers.org>

Copyright 2007-2015 Roger Meier
