# Supplementary material for: A Lab Assembled Microcontroller-Based Sensor Module for Continuous Oxygen Measurement in Portable Hypoxia Chambers
Source: PLoS One. 2016 Feb 10;11(2):e0148923. doi: 10.1371/journal.pone.0148923 (PMC4749204; doi:10.1371/journal.pone.0148923)
Supplement: S2 Table — (DOCX) [file pone.0148923.s004.docx]

**S2 Table. Stepwise instructions for initiating data collection in CoolTerm program.**

1. Connect the Arduino board to the computer to allow the USB port be recognized.
2. Open the Arduino program (IDE) and upload the Arduino sketch to the Arduino board.

3. To collect data, open the CoolTerm program and edit the default settings to those listed in **S1 Table**.

4. Set CoolTerm to collect initial reads at 1 sec intervals using the following commands: **Connection⮱Capture to text file⮱Start**

5. The program will suggest a file name of the format "CoolTerm Capture (date)(time)" to save incoming sensor data. Accept the default file name and "Save" to a preferred folder in the computer. Then, select "**Connect**" to start capturing data at the default rate of 1 read sec^-1^.

6. Switch the data collection rate to 1 read every 5 min by using the following commands: **Connection⮱Send String⮱(•ASCII)⮱”type” m⮱Send**.

7. CoolTerm will start recording at 5 min intervals (Fig. 4).

8. To read at 1 sec intervals again, simply reset the port using the following sequence: **⮱Connection⮱Reset Port**, and the reads will default to 1 sec intervals.

9. At the end of the experiment (48-72 hrs), save the CoolTerm data file:

**⮱Connections⮱Capture to text file⮱Stop**. Each data string from the oxygen sensor consists of 41 bytes. Thus, ASCII data collected during a 72 hr experiment can easily fit within a 100 kilobyte program space in CoolTerm.

To re-purge the hypoxia chamber mid-experiment (i.e., when O_2_ level increases above a threshold) to lower the O_2_% back to a desired level, a “reset” command in CoolTerm **(⮱Connection⮱Rest Port**) will default the reads to 1 second intervals. The researcher can then connect the gas mixture tubing via the flow meter at a low-flow rate (at 5 L/min) to the hypoxia chamber gas inlet (while the chamber is still in the 37 °C incubator), release the inlet and outlet clamps and monitor the drop in O_2_ tension until the desired level is reached. Then, the gas is shut off at the flow meter, inlet and outlet ports of the hypoxia chamber clamped, and monitoring continued again at 5 min intervals by giving an <**m**> command through the CoolTerm monitor (**Connection⮱Send String⮱(•ASCII)⮱”type” m⮱Send**).

If necessary, the Arduino sketch we have presented can be easily modified by a researcher to alter the data acquisition intervals. A simple text based alterations on two lines in the program code (as indicated below) is all that is necessary.

**delayPeriod = 300000; //if the char is m, set delay to 5 minutes – change this if necessary**

**delayPeriod = 1000; //otherwise, delay is set to 1 second – change this if necessary**
