# Supplementary material for: A Lab Assembled Microcontroller-Based Sensor Module for Continuous Oxygen Measurement in Portable Hypoxia Chambers
Source: PLoS One. 2016 Feb 10;11(2):e0148923. doi: 10.1371/journal.pone.0148923 (PMC4749204; doi:10.1371/journal.pone.0148923)
Supplement: S1 Text — (DOC) [file pone.0148923.s005.doc]

**S1 Text. The Arduino Sketch^a^ for data transfer and serial communication^b^.**

**_____________________________________________________________________________________________**

**/***

**Receives from software serial port, sends to hardware serial port.**

*** RX is digital pin 10 (connect to TX of level shifter: pin B1)**

*** TX is digital pin 11 (connect to RX of level shifter: pin B2)**

**The code was written for Arduino Uno Rev. 3 and Arduino IDE ver. 1.6.1.**

***/**

**#include <SoftwareSerial.h> //incorporate software-serial library**

**SoftwareSerial myserial(10, 11); //enable software serial port to RX,TX**

**long delayPeriod; //introduce “delayPeriod”**

**String Luminoxstring = ""; //string to hold incoming data from Luminox-O2 sensor**

**boolean Luminox_stringcomplete = false; //were all data from Luminox-O2 sensor received? Check**

**void setup()**

**{**

**Serial.begin(9600); //set baud rate for Arduino serial port to 9600**

**myserial.begin(9600); //set baud rate for software serial port to 9600**

**Luminoxstring.reserve(41); //set aside 41 bytes for receiving data from Luminox-O2 sensor**

**}**

**void serialEvent()**

**{**

**String inchar = "";**

**while (Serial.available() > 0) { //while a char is held in software serial buffer**

**inchar += (char)Serial.read(); //grab that char**

**if (inchar == "m")**

**delayPeriod = 300000; //if the char is m, set delay to 5 minutes – change this if necessary**

**if (inchar == "s")**

**delayPeriod = 1000; //otherwise, delay is set to 1 second – change this if necessary**

**}**

**}**

**void loop()**

**{**

**{ //start of loop sequence**

**while (myserial.available()) { //while a char is held in software serial buffer**

**char inchar = (char)myserial.read(); //grab that char**

**Luminoxstring += inchar; //add the received char to LuminoxString**

**if (inchar == '\r') { //if the incoming character is a <term>, reset**

**Luminox_stringcomplete = true;**

**}**

**}**

**if (Luminox_stringcomplete){ //was a complete string received from the Luminox sensor?**

**Luminoxstring.remove(41); //remove any serial string overruns**

**Serial.print(Luminoxstring); //use the Arduino serial port to send that data to CoolTerm**

**Luminoxstring = ""; //clear the Luminoxstring:**

**Luminox_stringcomplete = false;**

**delay(delayPeriod); //5 minutes(300000 millis); or 1 second (1000 millis)**

**}**

**}**

**}**

______________________________________________________________________________________________________________________________________

^a^ (copy and paste the above program text into Arduino IDE as a new sketch).

^b^ Open-source Arduino examples (Arduino.cc) for "SoftwareSerial"(Reference / SoftwareSerial),"SerialEvent"(Tutorial / SerialEvent), and "String.reserve" (Reference / StringReserve) were used in generating code. A "StringRemove" "(Tutorial / StringRemove) command was embedded in the code to eliminate data string overruns that occur when a sampling rate of >2000 mSec is used, which can result in staggered text output from the CoolTerm program.
